# Supplementary figures and images for: A fungal lytic polysaccharide monooxygenase is required for cell wall integrity, thermotolerance, and virulence of the fungal human pathogen Cryptococcus neoformans
Source: PLoS Pathog. 2023 Apr 26;19(4):e1010946. doi: 10.1371/journal.ppat.1010946 (PMC10166503; doi:10.1371/journal.ppat.1010946)

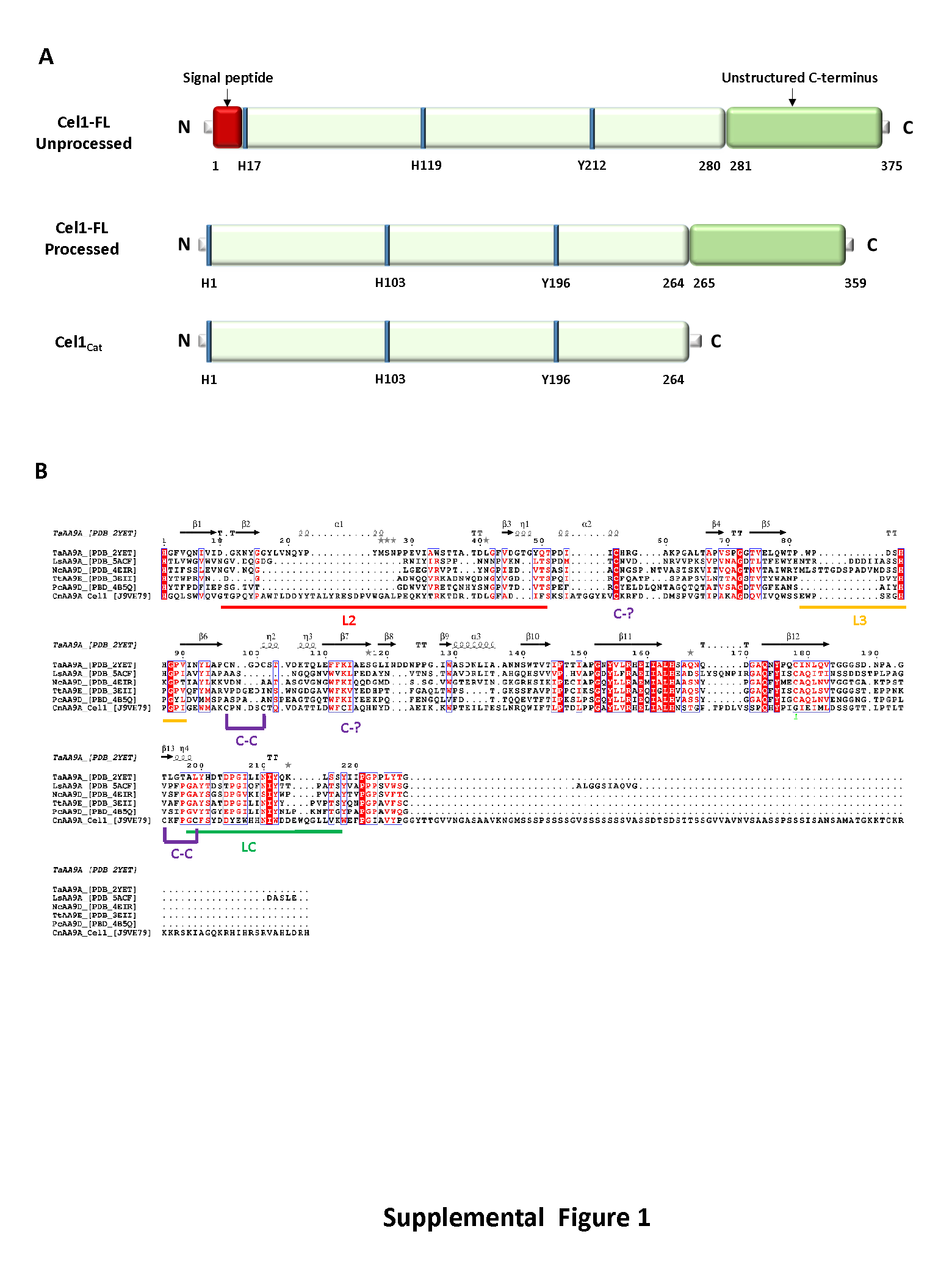

Supplement: S1 Fig — (A) Schematic domain overview of CnCel1 protein variants. The N-terminal signal peptide is highlighted in red, putative copper binding amino acids are highlighted in blue and labelled, and the unstructured C-terminus is highlighted in dark green. (B) Structure based alignment of Cel1 and the AA9 sequences from the phylogenetic analysis. The alignment was created using EXPRESSO of the T-COFFEE alignment package [85]. The final alignment was prepared using the ESPript 3.0 web server [90]. The substrate binding regions, L2, L3, and LC, are indicated, as well as the two paired cysteine disulfide bridges and two unpaired cysteines from the two protein fold models. (TIF) [file ppat.1010946.s001.tif]

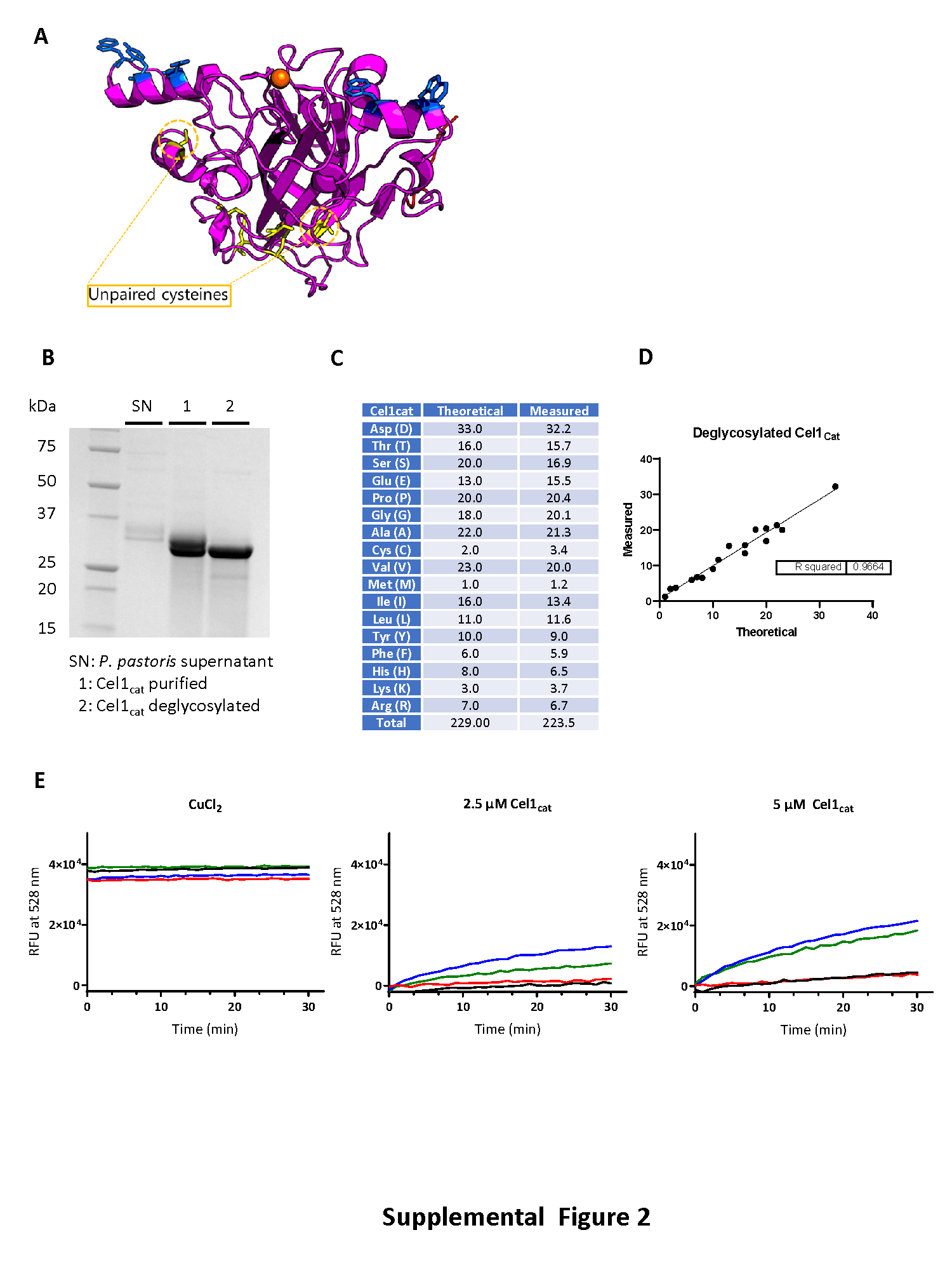

Supplement: S2 Fig — (A) Alphafold2 model of CnCel1, turned 180 on the y-axis compared to Fig 1B. CnCel1 is shown in magenta. CnCel1 aromatic residues potentially involved in substrate binding are shown as stick representation in blue and CnCel1 cysteines in yellow. The two unpaired cysteines are indicated. (B) Representative coomasie stained polyacrylamide gel of the expression supernatant, purified, and deglycosylated Cel1Cat protein. (C-D) Quantitative amino acids acid analysis of deglycosylated Cel1Cat confirms expression of correct protein. (E) Progress curves for oxidation of reduced fluorescein by non-enzyme bound copper and purified Cel1cat without added copper. Activity measured with relative fluorescent units (RFU) at 528 nm for 30 min. Free CuCl2 was tested as a negative control in stoichiometric amounts to a 5 μM copper loaded Cel1cat (3.75 μM). Cel1cat concentrations as indicated, without copper loading, was tested to assess initial copper loading from expression host. Reaction conditions are 75 mM phosphate citrate, pH 7.4, 25°C, Cel1cat blank (black line), 100 μM DHA (red line), 100 μM H2O2 (blue), or both (green line) was added to investigate LPMO copper reduction. Experiments done in triplicates, standard deviations shown, but not visible. (TIF) [file ppat.1010946.s002.tif]

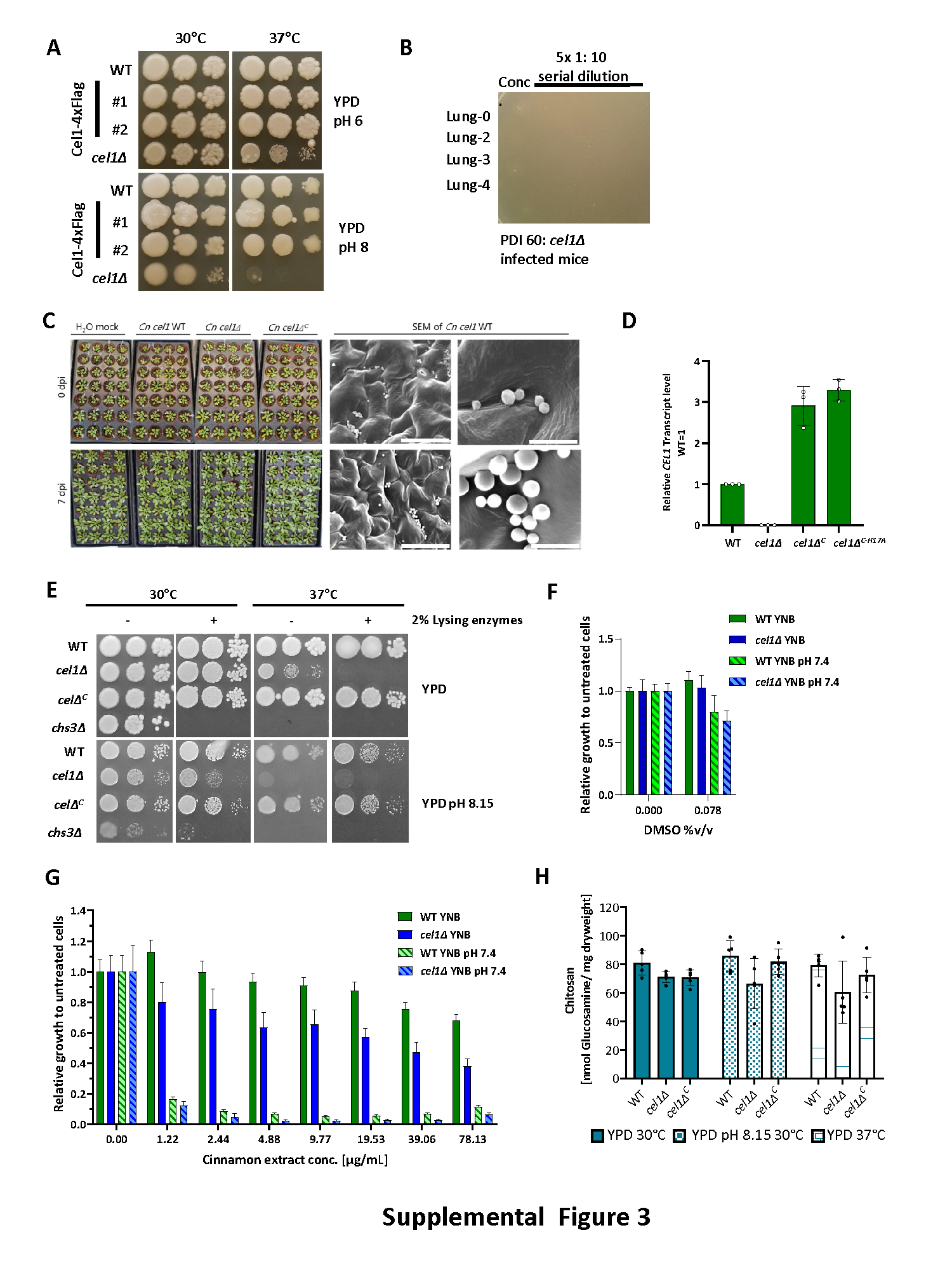

Supplement: S3 Fig — (A) Phenotypic analysis of the Cel1-4xFLAG strain. The WT, cel1Δ mutant, and two Cel1-4xFLAG expressing strains were spotted in serial dilutions on YPD or YPD pH 8.15 medium and incubated for 3d at indicated temperatures. (B) Spotting based lung CFU analysis from lungs lysates 60 days post infections. Lungs were harvested from mice infected with the cel1Δ strain. Concentrated as well as a serial dilution of the harvested Lung lysate was spotted on YPD-Chloramphenicol and incubated for 3d at 30°C. (C) Plant infection analysis. 28 three week-old, soil-grown Arabidopsis thaliana col-0 plants were drop inoculated with either sterile water, WT, cel1Δ, or the cel1ΔC strains. Representative images are shown of plants at the inoculation day (0 days post inoculation, dpi) and 7 dpi. Leaf samples infected with the WT strain were collected for scanning electron microscopy to examine fungal phenotype and behavior. White scale bar illustrates 50 μm for the right column and 10 μm for the left column, respectively. (D) Transcript analysis of CEL1 abundance in cel1ΔC-H17A and other indicated strains after a 3h induction in SC pH8.15 at 30°C. Quantitative RT-PCR was used to assess relative CEL1 transcript levels for each strain compared to the WT strain. Presented is the mean +/- SEM of the relative transcript levels of 3 biological replicates. (E) Five-fold serial dilutions of cell suspensions for each strain were incubated on YPD or YPD pH 8.15 at 30°C or 37°C for 3d, in the presence or absence of lysing enzymes extracted from Trichoderma harzianum. (F) Inhibition growth control of the highest DMSO concentration used in the MIC analysis at both pH conditions. Growth inhibition reported as in the MIC graph (G). (G) Minimal inhibitory concentration (MIC) analysis of cinnamon extract, dissolved in 50% DMSO, in unbuffered YNB and host condition-buffered YNB (YNB-pH 7.4). Cn WT and cel1Δ strains were cultivated in 96-well liquid cultures and treated with 2-fold serially diluted cinnamo [file ppat.1010946.s003.tif]

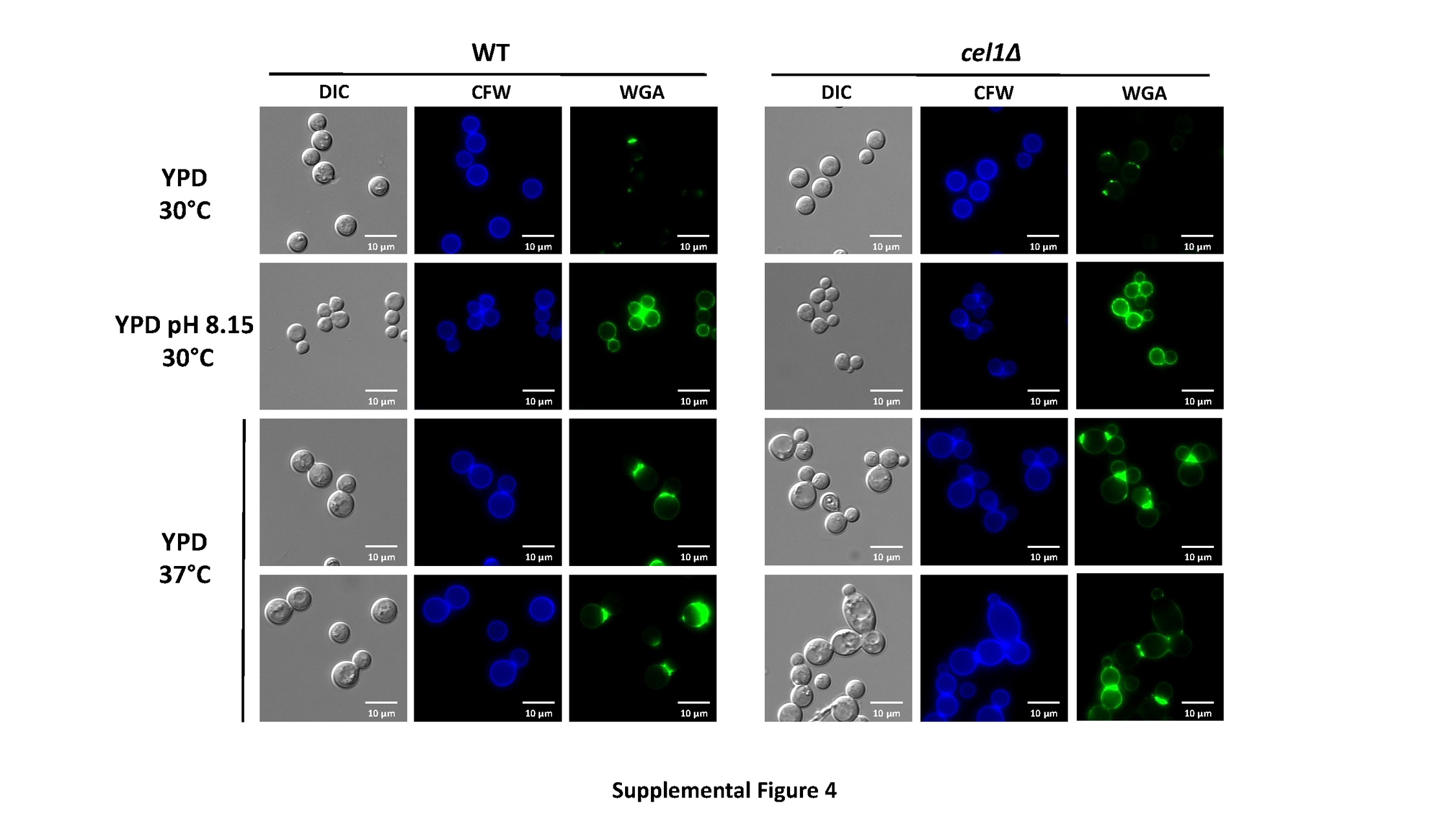

Supplement: S4 Fig — The WT and cel1Δ strains were incubated overnight in YPD medium and resuspended to OD600 of 0.1 in indicated medium (either YPD or YPD pH 8.15). Cells were cultivated for 24h hours at indicated temperatures and stained with CFW (total cell wall chitin) and WGA-Alexa488 (exposed cell wall chitin). Shown are the single channel images of CFW (blue channel), WGA-Alexa-488 (green channel) used for creating the merged images with Image J/Fiji as well as the corresponding DIC image of this analysis. (TIF) [file ppat.1010946.s004.tif]

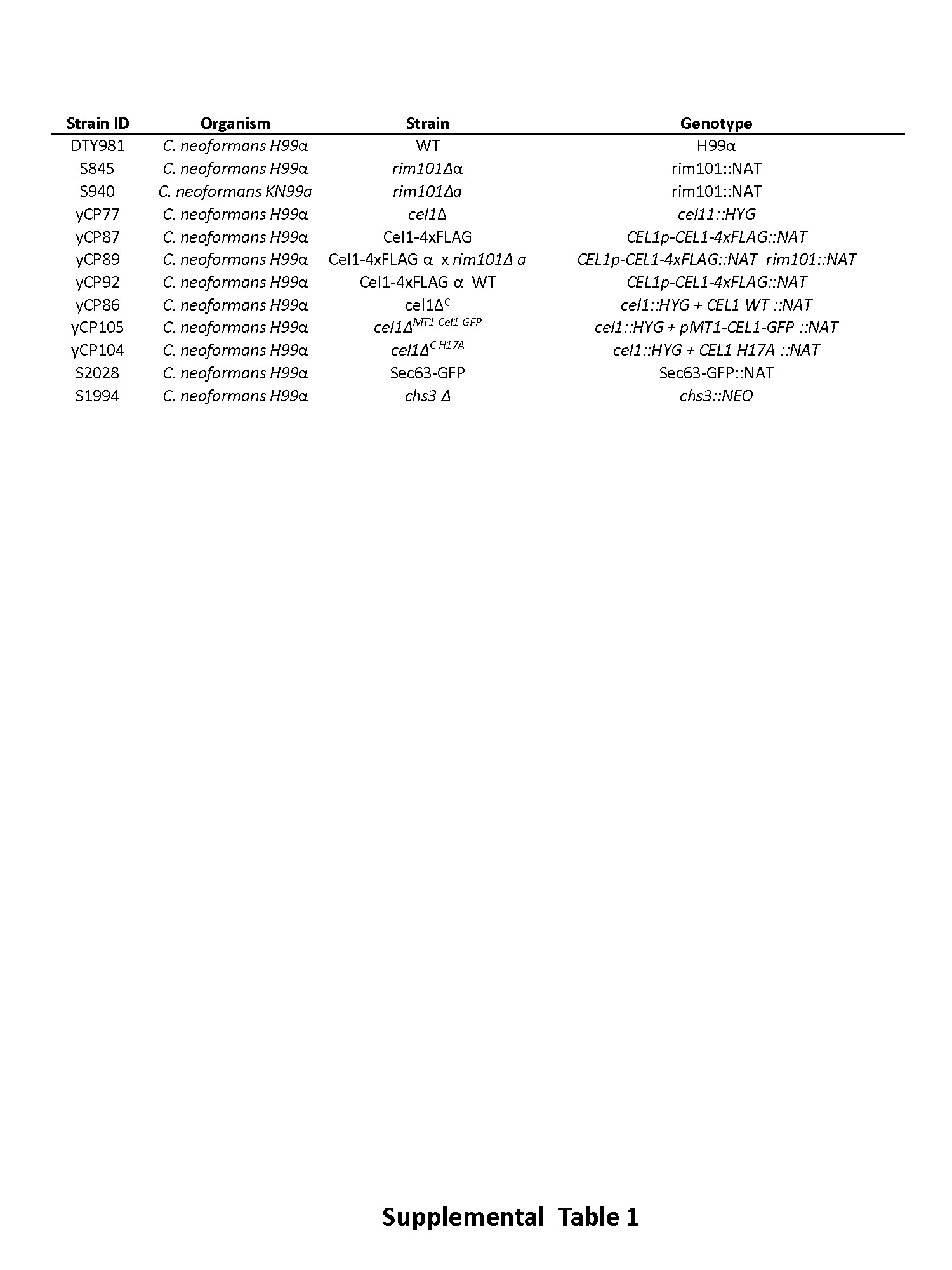

Supplement: S1 Table — (TIF) [file ppat.1010946.s005.tif]

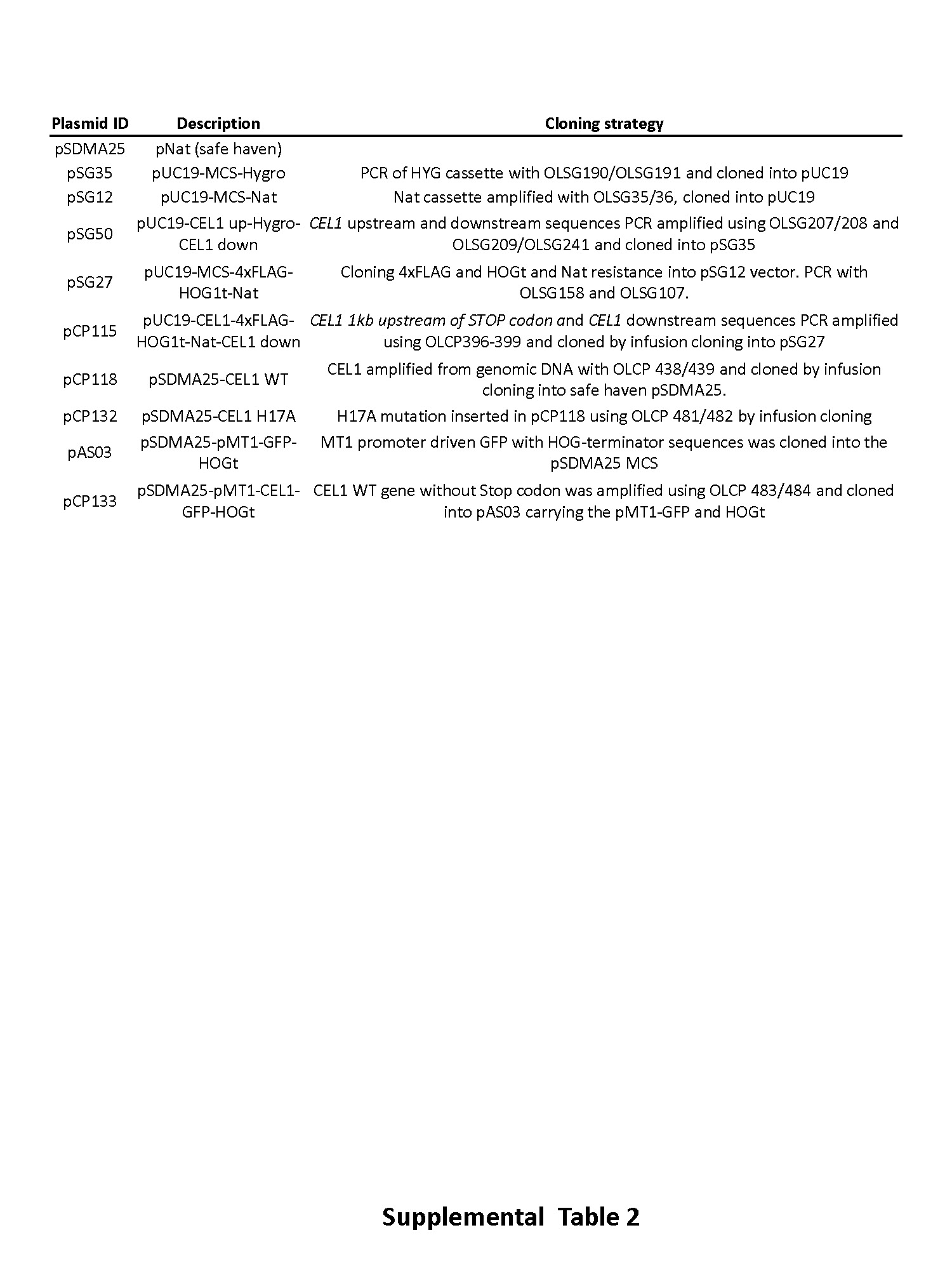

Supplement: S2 Table — (TIF) [file ppat.1010946.s006.tif]

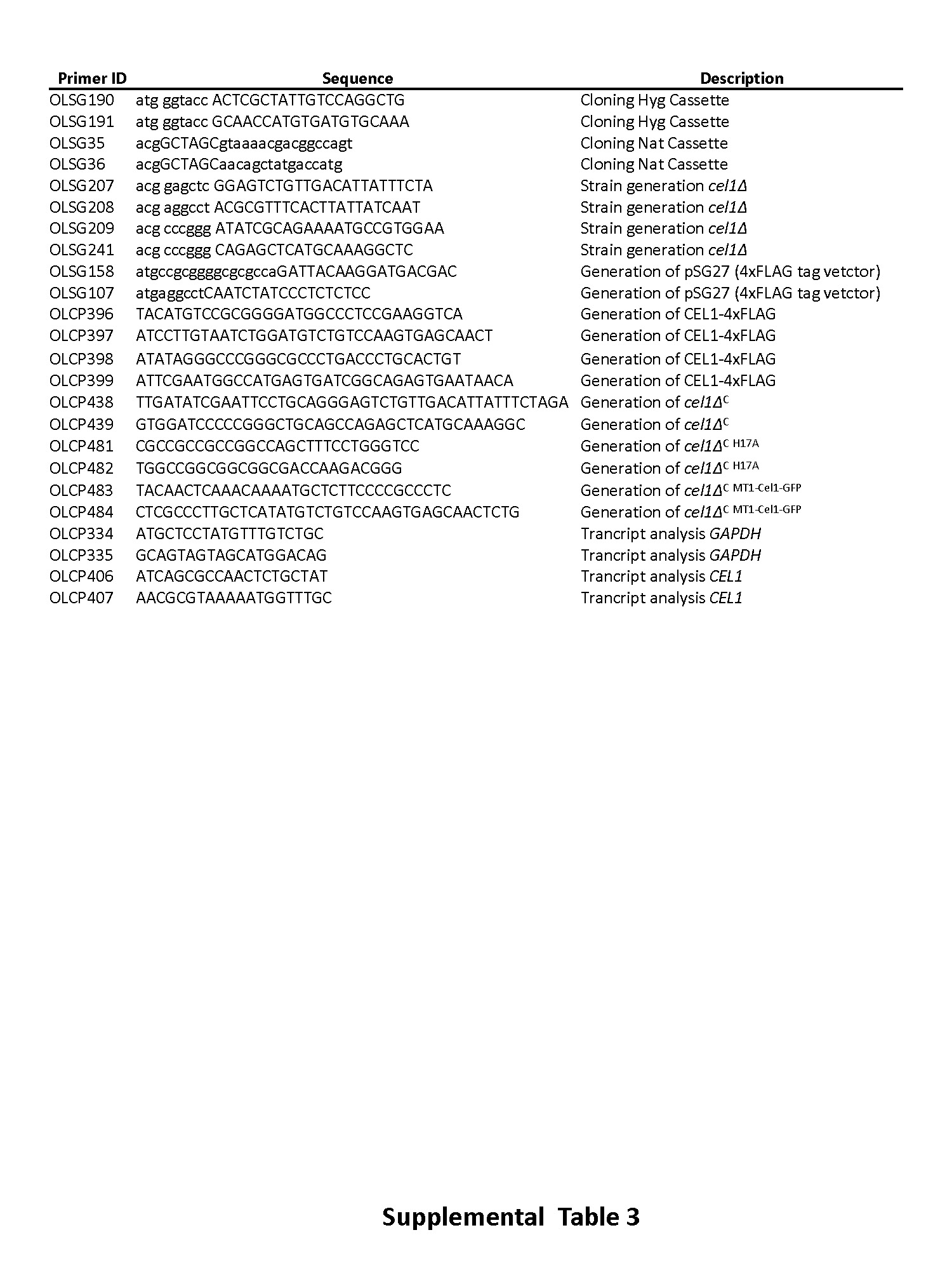

Supplement: S3 Table — (TIF) [file ppat.1010946.s007.tif]
