# Supplementary material for: A fungal lytic polysaccharide monooxygenase is required for cell wall integrity, thermotolerance, and virulence of the fungal human pathogen Cryptococcus neoformans
Source: PLoS Pathog. 2023 Apr 26;19(4):e1010946. doi: 10.1371/journal.ppat.1010946 (PMC10166503; doi:10.1371/journal.ppat.1010946)
Supplement: Attachment — Submitted filename: 20230320_ Response to reviewers_final.docx [file ppat.1010946.s008.docx]

**Response to reviewers**

**(PPATHOGENS-D-22-01837) - [EMID:194c9bcb1e2526fd]**

**Summary:** We thank the editor and reviewers for their very insightful comments. We have attempted to respond to all of the comments from the prior reviews. The two biggest issues identified for revision included further defining the site of cellular localization for the *Cn*Cel1 protein, and better characterizing its function as an enzyme, including its likely substrates. We have performed additional experiments to address these issues. Importantly, the N- and C-termini of the *Cn*Cel1 protein are important for its function, and the creation of simple fusion proteins with fluorophores at these sites is not feasible while preserving protein function. We therefore used enzymatic digestions of the fungal cell wall, and subsequent release of the *Cn*Cel1 protein, as further supporting evidence of its association with the fungal cell surface.

Second, there were questions about the nature of the assay demonstrating that *Cn*Cel1 indeed functions like an LPMO in terms of its enzymatic activity. A manuscript, written by a subset of our authors, was recently accepted for publication that describes the functional assay that we use here to suggest LPMO-like enzymatic activity (see below). Additionally, we have tested the purified *Cn*Cel1 protein on several standard LPMO substrates as suggested by the reviewers, and none were convincing substrates. A statement to this effect was added to the manuscript. However, the data suggest an important and perhaps unique substrate for *Cn*Cel1 that will take careful and more detailed experimentation to precisely define.

Lastly, the reviewers asked for more detail regarding antifungal activity for compounds previously shown to inhibit LPMO function in other systems. The cinnamtannin B compound, isolated from cinnamon extracts, was chosen for its inhibitory activity against specific LPMOs that are distantly related to *Cn*Cel1. However, in this revised manuscript, we do demonstrate that the same extracts have very potent antifungal activity against *C. neoformans* growth. Importantly, this growth inhibition is only observed in condition in which the *Cn*Cel1 protein is expressed, and not in rich media at 30C.

Together, the reviewer comments have allowed us to create a clearer experimental narrative, a much-improved manuscript, and a clearer translational story. We have prepared a detailed response to all comments from the recent review.

**Major Issues:**

**Reviewer #1: 1.Line 97. The hypothesis to justify focusing on LPMOs (endogenous fungal substrates) is presented, but the substrates are not identified in the initial section of the Results (e.g., lines 146-155; Discussion line 420-421). The manuscript would be stronger if substrates were identified and linked to the subsequent focused analysis of the cell wall**.

*Response:*

We agree the manuscript would be stronger with an identified substrate. As described in the revision, we have carried out initial screening on isolated polysaccharides (avicel, PASC, beta-glucan, xylan, arabinoxylan, chitin, chitosan, and glucomannan) as common substrates for other LPMOs. However, these data remain inconclusive, and none are convincing individual substrates. A more thorough biochemical characterization and purification of more *in vivo* relevant substrates is beyond the scope of study. We show that the protein is indeed an enzyme with copper dependent redox activity, as has been demonstrated for several other LPMOs and something that differentiates *Cn*Cel1 from the LPMO-like protein *Cn*Bim1/Cbi1. The methodology used to support *Cn*Cel1 as an enzyme were recently published by a subset of the authors of the current manuscript. This publication allows a more rigorous assessment of the enzymatic potential of *Cn*Cel1, and it hopefully addresses the reviewers’ justified requests for more information about this assay.

**2. Line 162, Fig. 2A. The expression of the flag-tagged protein is remarkably strong and specific for the pH 8 condition and no signal (even a weak one) is seen for the other conditions/lanes. Can the authors comment on why the expression is so specific?**

*Response:*

As we demonstrate in this paper, the *CEL1* transcript is regulated by the pH-responsive Rim101 transcription factor. In prior studies, Rim101 has been demonstrated to control the expression of many *C. neoformans* genes in a pH-dependent manner. This transcription factor itself is cleaved and activated in a “dose-responsive” manner as the pH increases from 6 to 8. The expression of the Flag-tagged *CEL1* allele (expressed under control of its own promoter) is therefore specifically induced at elevated pH consistent with its regulation by Rim101. Accordingly, no pH-dependent transcript induction of *CEL1* is observed in a *rim101*Δ mutant strain at alkaline pH. We have attempted to clarify this issue by changes in the Discussion where we discuss the role of Rim101 in *CEL1* expression, similar to other genes encoding cell wall and capsule modifying enzymes.

**3. Line 169. No expression is seen in the rim101 mutant. However, in Fig. 5A there is a difference in the phenotypes for the rim101 mutant versus the cel1 mutant at 37C – e.g. on SDS, CFW, caffeine. What is the explanation for this difference – the media conditions for the experiments?**

*Response*:

We agree that this was not clear in the original version. We have offered potential explanations for differences between the *rim101*Δ and *cel1*Δ mutant phenotypes under different stress condition as follows:

“The differences in phenotypes between the *cel1*Δ and *rim101*Δ strains during infection and *in vitro* stresses confirm prior transcriptional analysis that *CEL1* is only one of several cell wall modifying enzymes regulated by the Rim101 pathway in response to changing environmental conditions.”

**4. Line 193. Is the cel1ΔMT1-Cel1-GFP construct functional?**

*Response*:

In response to the reviewers request for us to better define the site of *Cn*Cel1 protein localization, we have modified the paper significantly.

First, in light of new data, we have elected to remove the experiments using the Cel1-Gfp constructs. We are unable to add an epitope tag to the N-terminus of *Cn*Cel1 since the first amino acid after protein cleavage is the histidine involved in the enzymatic function. Although we demonstrate that the addition of small epitope tags to the C-terminus allows the protein to retain function (as documented by complementation of mutant phenotypes), the addition of the very large GFP moiety appears to compromise *Cn*Cel1 function. When we explored this further, we determined that, unlike the C-terminal FLAG-tagged Cel1 protein, Cel1-Gfp is retained inside the cell and does not localize to the cell wall.

Second, we have instead focused on enhanced experiments to support cell wall/cell surface localization of the Cel1-FLAG fusion protein that is functional as assessed by complementation of mutant phenotypes (see below).

**5. Line 196. The evidence is not clear for the conclusion in title for Fig. 3 that trafficking occurs by the ER/Golgi route – a more detailed analysis would be needed to support this conclusion - eg. BFA treatment. The images in Fig. 3C are a bit murky (especially the DAPI) and it would help to have DIC images of the cells.**

*Response*:

We agree, and these issues are now addressed by our removal of the Cel1-Gfp data.

**6. Lines 246-261. This section reports negative results and seems strangely uninformative about Cel1 given that the wt strain did not cause symptoms. Why would one expect yeast cells to penetrate the plant surface? In general, I think this section detracts from the paper and could just be mentioned as a brief statement.**

*Response*:

We agree that these are primarily “negative data”. We included them given the importance of LPMOs in plant fungal pathogens in the establishment of colonization. Given the varying reports in the literature of *C. neoformans* association with live plants, we think that the role of *C. neoformans* LPMOs on plant colonization/invasion will be a very natural question for many readers. Therefore, we have chosen to include an abbreviated description of these results in the revised version, in accordance with the reviewer suggestions.

**7. Line 295. Is it known that the mutated Cel1-H17A protein was actually expressed?**

*Response*:

In the revised version, we have included data from quantitative rt-PCR experiments in which we demonstrate a very similar level of expression of the *CEL1-H17A* allele compared to the re-integrated *CEL1-WT* allele.

**8. Lines 310-311, Figure 5C. It was difficult to appreciate that the evidence from the figure justified the conclusion. It would help to have DIC images for clearly identifying the multiply budded cell sites, and perhaps a more detailed analysis of staining would demonstrate the difference. Could this be quantified by flow cytometry?**

*Response*:

We agree with the reviewer. We have added a new Sup Figure 4 to the MS which shows the single channel images used to create Figure 4C.

**9. Lines 338-350. In general, my examination of Fig. 6B,C,D and F suggests not obvious difference for the capsule. The enlarged cell body is the only convincing result.**

*Response*:

We have repeated these experiments and revised the image with a new panel (with a longer time of electrophoresis) to more clearly demonstrate the difference in electromobility of the secreted capsule in the *cel1∆* strain.

**10. Lines 392-396. This first paragraph contains a jumble of ideas (especially the first sentence) and could be deleted.**

*Response*:

We have removed this paragraph without detracting from the flow of the Discussion.

**11. Line 537. Did the authors consider testing cinnamtannin B1 (if available) with C. neoformans?**

Response:

We have included MIC experiments with cinnamon extract, from which cinnamtannin B1 was purified, these data were added to Supp Figure 3. These experiments demonstrate a very potent growth inhibition only in conditions in which the *Cn*Cel1 protein is expressed.

**Reviewer #2:**

1) **Although the authors have the enzymatically active recombinant protein, they did not test this against any candidate substrates of LMPO, e.g., chitin or other polysaccharides in the cell wall or capsule. Even a negative result would help to pinpoint the mechanism by ruling out possibilities.**

*Response:*

In the revised manuscript, we have added a statement that we were unable to document enzymatic activity against a panel of standard LPMO substates using the purified recombinant protein. We are currently using a variety of other approaches to identify native *C. neoformans* compounds that may act as novel or less conventional substrates for *Cn*Cel1. Given the rigor required for these experiments, we propose that they are beyond the scope of the current manuscript. However, we have now included the “negative result” suggested by this reviewer, an important addition.

**2) The localization studies were conducted with a protein greatly over-expressed under Cu toxicity stress conditions. They find much of the protein in the Golgi and ER. Is it being trapped there because of the over-expression and/or Cu stress conditions?**

**The authors claim there is some cell surface localization, but Cel1 localization looks indistinguishable from the control Sec63 that is ER/Golgi. If Cel1 is expressed at physiological levels, would it largely show localization at the cell surface where it can modify the capsule or cell wall material? The accurate localization of this protein would certainly shed important light into the mechanism of action.**

*Response*:

Please see response to similar comments from Reviewer 1. We have removed the experiments using the Cel1-Gfp allele given our subsequent documentation of its lack of function and likely altered localization by the large Gfp-moiety. We have included additional experiments using a functional Cel1-FLAG protein in which enzymatic digestion of the cell wall results in a dose-dependent removal of Cel1 from being associated with the fungal cell. In these experiments, we documented no off-target general protease activity of zymolyase that would have confounded the interpretation of these results.

**Reviewer#3: 

I. The authors show that Cn CEL1 is an outlier in the AA9 group and that the patterns of oxidation Cel1 catalyzes are quite different from those of a control AA9 protein (Fig. 1D). Are they certain it has the attributed activity? This figure is also central to the conclusions of the paper, so enough information must be provided for the reader to understand and evaluate these results. For example, the text for Fig. 1D should include the rationale for adding ascorbate, hydrogen peroxide, or both. It would also be helpful to know why fluorescein was used instead of the substrate characterized in the cited paper (22).**

Response:

The assay for LPMO redox activity was optimized using dehydroascorbate (not ascorbate). A detailed description of the development of the assay has now been accepted for publication as indicated by the new reference that has been added to the text. The addition of this publication as a citation in the current manuscript greatly adds to the ability to interpret our results supporting the LPMO-like activity of the purified *Cn*Cel1 protein. The fluorescein assay has the advantage of being kinetic (continuous) whereas the previously assay required the enzyme reaction to be stopped with base at different time points to develop color of the product.

**II. Despite having achieved expression and purification of an active protein, the authors delve no further into the biochemical function of Cel1. Given the major questions about wall synthesis that (as they point out) remain to be answered, this seems like a missed opportunity. Further analysis might also address the previous point.**

Response:

A thorough biochemical analysis of *Cn*Cel1 and identification of substrate is beyond the scope of this MS and will be addressed in an additional more enzymatically focused MS. Also, see answer to reviewer #1 and 3

**III. Central to the authors’ conclusion is that Cel1 remains at the cell surface. The very nice images in Fig. 3C clearly show that Cel1-GFP occurs in the secretory pathway, but this could also occur if it was en route to release from the cell. The key information, therefore, is the difference between the cellular distribution of Cel1-GFP and the Sec63-GFP control. While the first image presented for Cel1-GFP appears to show puncta on the cell surface that are not present with Sec63-GFP, the second Cel1-GFP image looks much like the control. The authors should quantitate surface versus internal puncta or otherwise further support this key critical conclusion. The Discussion should also include how the authors think Cel1 remains associated with the cell surface.**

*Response*:

Please see response to similar comments from Reviewer 1 and 2. Many of these suggested experiments are no longer needed given our removal of the Cel1-Gfp data.

Other issues that would potentially require experimentation (with line number):

**311 The statement about WGA staining intensity is not supported by the images shown and should be modified or supported by additional data.**

*Response*:

We agree, and we have changed the text accordingly.

“However, the *cel1*Δ strain exhibited a notable change in budding behavior at high temperatures compared to WT, including an increased number of cells with multiple buds and wide bud necks (Fig 4 C, Supp Fig 4).”

**348 Fig. 6F is hard to interpret because of apparent variability in this method. For example, the first and third lanes look more similar than the first and second. Although the mutant material does run slightly higher on the gel, its most intense region, at least in lane three, is lower than WT. Overall, the conclusions about this experiment do not seem well supported and should be modified or supported by additional data.**

*Response*:

We have repeated those experiments and now offer a new image (with a longer period of electrophoresis) that more clearly demonstrates a difference in electromobility of the secreted capsular polysaccharide in the *cel1∆* strain.

**Part III – Minor Issues: Editorial and Data Presentation Modifications**
Please use this section for editorial suggestions as well as relatively minor modifications of existing data that would enhance clarity.

**Reviewer #1: I focused on key issues, as presented in Part II.**

**Reviewer #2:**

**1) Previous studies by Thiele et al described a LMPO like protein Bim1 that is important for Cu homeostasis but there is no mention of this in the introduction.**

*Response*:

We agree that mentioning the Bim1 protein would be clarifying. Since the initial report identifying *Cn* Bim1, we and our collaborators have subsequently determined that the Bim1/Cbi1 protein does not possess LPMO-like activity and plays a very distinct role in cell wall and copper homeostasis. We have included this information in the revised Introduction, with recent citations included.

**2) In Fig. 2A the authors only tested one dose of BCS to conclude Cel1 is not regulated by Cu starvation. Is there a control to show that this dose creates a Cu starvation stress state in the yeast?**

*Response*:

We used BCS and Cu concentrations which previously have shown a strong Cu-stress response in *Cn* (Garcia-Santamarina et al., 2018).

**3) Is the control localization of Sec63 in Fig. 3 also done under Cu toxicity stress conditions?**

*Response*:

This issue was addressed by our removal of the Cel1-Gfp data.

**4) The studies attempting to show fungal virulence in a plant model (without success) seem beyond the scope of this paper.**

*Response*:

Please see response to similar comments from Reviewer 1.

**Reviewer #3:**

**81 How carbohydrates might be ‘recalcitrant’ should be explained.**

*Response*:

We agree, and we have changed the text accordingly.

**116 The authors should mention why they narrowed their focus to 00601.**

*Response*:

We agree, and we have changed the text accordingly.

**130 What constitutes ‘acceptable’ should be explained.**

*Response*:

We agree that this was unclear wording, and we have removed this word.

**145 What stain is used in Supp Fig 2B?**

*Response*:

A Coomassie stain was used. We added this information to the figure legend.

**161 ‘Phenotypically identical’ is an overstatement given that growth in only two conditions is tested.**

*Response*:

We agree, and we have changed the text accordingly.

“This strain was phenotypically similar to the wild-type strain, and not to the *cel1*Δ mutant strain, in all tested *in vitro* conditions, indicating that this fusion protein was likely fully functional (Supp Fig 3A).”

**180 The differences in protein expression at various temperatures are modest, and the amount at 37° seems slightly less than at either 30° or 39°.**

Response:

Even though the difference between 30°C and 37°C is only modest and hard to quantify, there is a clearly visible increase of the Flag-HRP signal from lower to higher growth temperature in the western blot shown.

**210 Although it does not change conclusions about Cel1, it is surprising that all mice did not succumb to WT/complement infection with the given inoculum.**

*Response*:

We agree. We chose to use the C57BL-6 mouse strain that is somewhat more resistant to *Cn* infection than many other commonly used mouse strains. We believe that the internal consistency of the current experiments supports the interpretation that is offered.

**272 Fig. 5A suggests the occurrence of suppressors. Have the authors looked into this?**

*Response*:

We agree that the appearance of colonies that grow well among the many colonies that are growth impaired suggest that suppressors my occur in this strain under this particular condition. The fact that suppressors do not occur in this strain in all stress conditions suggests that this is not a general phenomenon. We have not yet explored potential mechanisms of the appearance of these apparent suppressors.

**294 The legend to Supp. Fig. 3 has errors including panel lettering. Also, there is no reason to show phenotypes of transformants that were incorrect and not used in the study.**

*Response*:

We agree and have corrected in the revised version.
